# An Approach to Realizing Process Control for Underground Mining Operations of Mobile Machines

**DOI:** 10.1371/journal.pone.0129572

**Published:** 2015-06-10

**Authors:** Zhen Song, Håkan Schunnesson, Mikael Rinne, John Sturgul

**Affiliations:** 1 Department of Civil and Environmental Engineering, School of Engineering, Aalto University, Espoo, Finland; 2 Division of Mining and Geotechnical Engineering, Department of Civil, Environmental and Natural Resources Engineering, Luleå University of Technology, Luleå, Sweden; 3 School of Civil, Environmental and Mining Engineering, University of Adelaide, Adelaide, Australia; China University of Mining and Technology, CHINA

## Abstract

The excavation and production in underground mines are complicated processes which consist of many different operations. The process of underground mining is considerably constrained by the geometry and geology of the mine. The various mining operations are normally performed in series at each working face. The delay of a single operation will lead to a domino effect, thus delay the starting time for the next process and the completion time of the entire process. This paper presents a new approach to the process control for underground mining operations, e.g. drilling, bolting, mucking. This approach can estimate the working time and its probability for each operation more efficiently and objectively by improving the existing PERT (Program Evaluation and Review Technique) and CPM (Critical Path Method). If the delay of the critical operation (which is on a critical path) inevitably affects the productivity of mined ore, the approach can rapidly assign mucking machines new jobs to increase this amount at a maximum level by using a new mucking algorithm under external constraints.

## Introduction

Process control is the automatic control of the output of a process according to the pre-set target by adjusting related variables of the process based on rules and models. It has been routinely applied in plant-wide production in minerals processing. It can also be used to achieve mining objectives. However, mining engineers have not implemented process control on a mine-wide scale for underground mines. Mineral processing is normally described as being in a continuous state which can be expressed using partial differential equations (PDEs). Mining operations are normally implemented in a serial (e.g. drilling, bolting) and cyclic (mucking) process, which is comprised of many discrete-events and requires an iterative procedure to represent the operations. Therefore, the techniques of process control that have been proven and widely used in minerals processing are not immediately suitable for mining operations. There have been a few mining operations (e.g. conveying and hoisting) which have applied control systems, but these systems are on single machine level, but not directly concerned with the entire mining process.

It is conceptually more difficult to utilize the concepts of process control for underground mining than surface mining. Underground mining is often required to invest more on cable-based communication and positioning, instead of using the existing wireless and GPS techniques which are the inherent advantage of surface mining. Additionally, underground mining has more various discrete operations than surface mining. The process of surface mining can be monitored visually and even controlled institutively by changing the working rates and the amount of haulers at each open pit. There have been several commercial products available in the market, for surface mines to obtain optimal or near-optimal results. However, the process control of underground mining has not been that formed as in surface mining, because of the complexity of excavation and production phases of underground mines.

**Fig 1:**
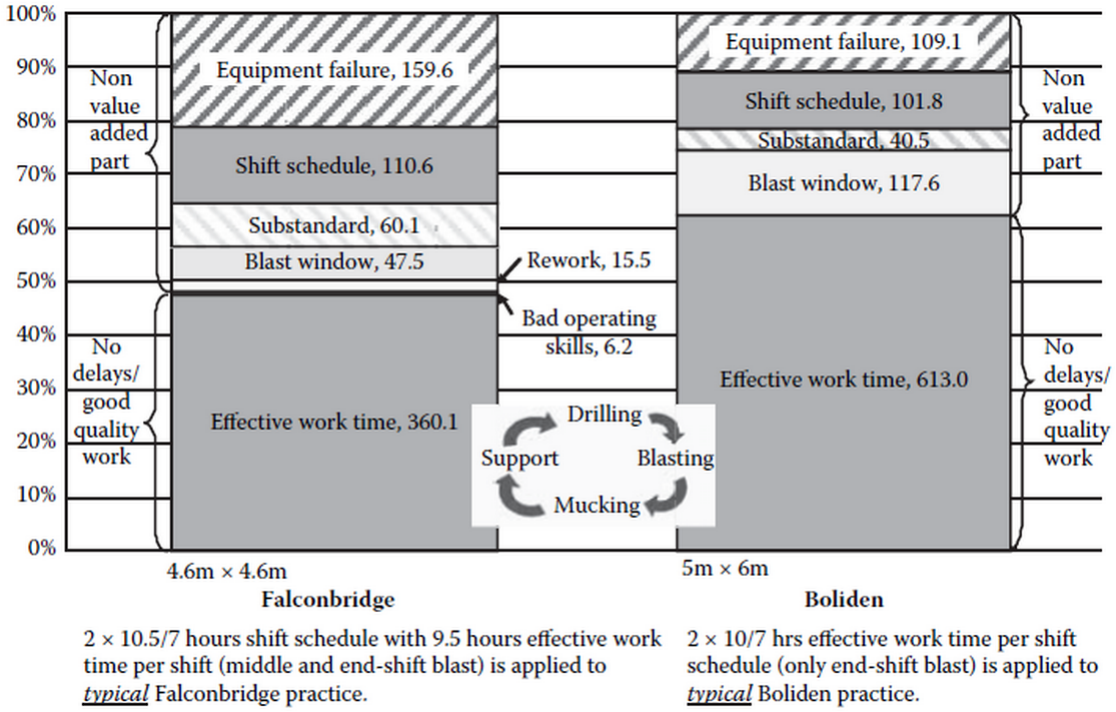
Time delay impacts for typical development practices (with working time in hour) with drilling and blasting [3].

Howes and Forrest [1] have applied short interval control to manage the underground mining process in Chelopech gold mine in Bulgaria. The mine aimed to double the production and achieve a 44% reduction in unit cost. A new approach was developed to manage shift tasks to achieve the production targets while improving the equipment utilization. Overcoming the underground communication barrier enables the mine to manage mining activities as well as detect any delays or interruptions during the shifts. The operation data collected during a shift have served as a current-state view to support work management, rather than just a historical reflection of past activities. The activities of the entire mine can be accessed through a monitoring, dispatching and coordinating center in the mine. It is also a source of information for miners in the field. Critical issues during a shift can be detected and corrected at earliest through the short interval control to reduce the potential loss of production. This approach eliminates the common paper-reporting procedure and improves the integrity and integration of data and information. The initial priority of this approach is to assist the shift management and work execution. In future, this approach will analyze trends and improve processes in a closed loop for a continuous improvement in the management of underground mining. Although Howes and Forrest’s work has practically realized the underground communication, integrated the mine-wide data, and established the efficient reporting system, there is still a need for an approach to control the process of underground mining operations of mobile machines. In manufacturing industry, the Lean Manufacturing and Six Sigma theories are mostly used in the management of product quality and work organization. Due to the very high uncertainties inherent in underground mining operations, it is not efficient and sufficient to only apply such theories in mining. Uncertainty has always been a factor in mining and many decisions traditionally rely on the experience and judgment of mine operators [2]. There was a process simulation analysis developed for a detailed analysis of the variables that impact the working time of excavation. The result of simulation analysis (Fig 1) was used to evaluate time delays and cost overruns in a specific process [3][4]. It shows that, even for modern mines in major mining companies, the shift schedule work is time-consuming, and can occupy a considerable percentage of daily work time.

In order to reduce the impact of personal experiences on the decision-making, and to improve the efficiency of the work process, it is required to develop an approach for the process control in underground mining. The approach should take the consideration of the uncertainty related to underground mining. The following sections describe the approach which includes an algorithm modified based on the existing PERT/CPM and an algorithm developed for mucking operation.

## New Algorithm for Serial Operations in Underground Mines

### 2.1 PERT and CPM

Program Evaluation and Review Technique (PERT) [5][6] and Critical Path Method (CPM) [7][8] are the first and so far the most prevailing scheduling techniques in project management. The PERT is a network-like scheduling technique, developed for the process control system for the development of the Polaris Missile Project. The Polaris project eventually included 23 PERT networks encompassing approx. 3000 activities. CPM is a computerized scheduling technique to improve the planning, scheduling, and reporting of DuPont’s engineering programs (including plant maintenance and construction projects). Normally both techniques are jointly used by the following steps:

1. Identify the specific activities to be scheduled;
2. Determine the specific sequence of the above activities;
3. Construct the network diagram;
4. Estimate the time required for each activity;
5. Determine the critical path;
6. Update the network diagram and the critical path along with the progress of the activities.

**Fig 2:**
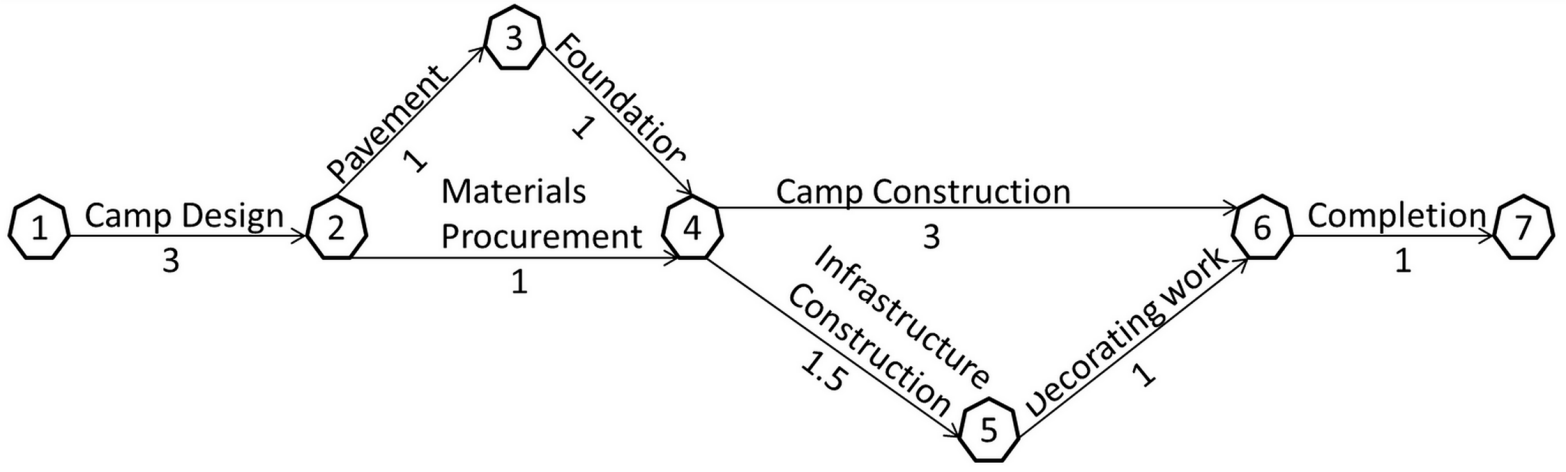
PERT/CPM used in an example of mine camp construction (with working time in week).

**Fig 3:**
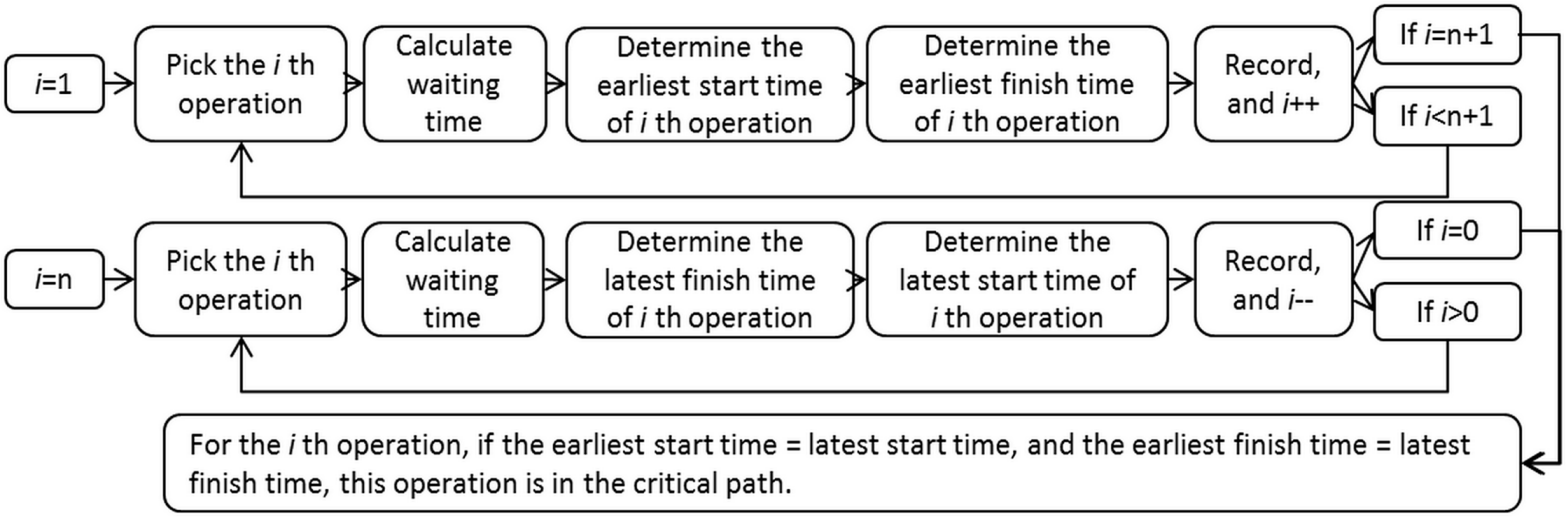
Flow chart of the conventional algorithm of determining the critical path.

Fig 2 shows a simplified example of managing the construction of a green-field mine’s camping area using the PERT/CPM. The activities and their durations are labeled on the both sides of arrow lines. The path with the longest duration of time from the start to the end is referred as the critical path. Any process cannot be completed sooner than the time accumulated on the critical path in the network. The critical path in this case is 1-2-3-4-6-7 with the total duration 9 weeks. The algorithm of determining the critical path is explained in Fig 3. The algorithm has two calculation processes, i.e. forward calculation and backward calculation. The forward calculation obtains the earliest starting time and earliest ending time of each activity, while the backward calculation obtains the latest starting time and latest ending time of each activity. If an activity has the same earliest starting time and latest starting time, and the same earliest ending time and latest ending time, this activity is in the critical path.

**Fig 4:**
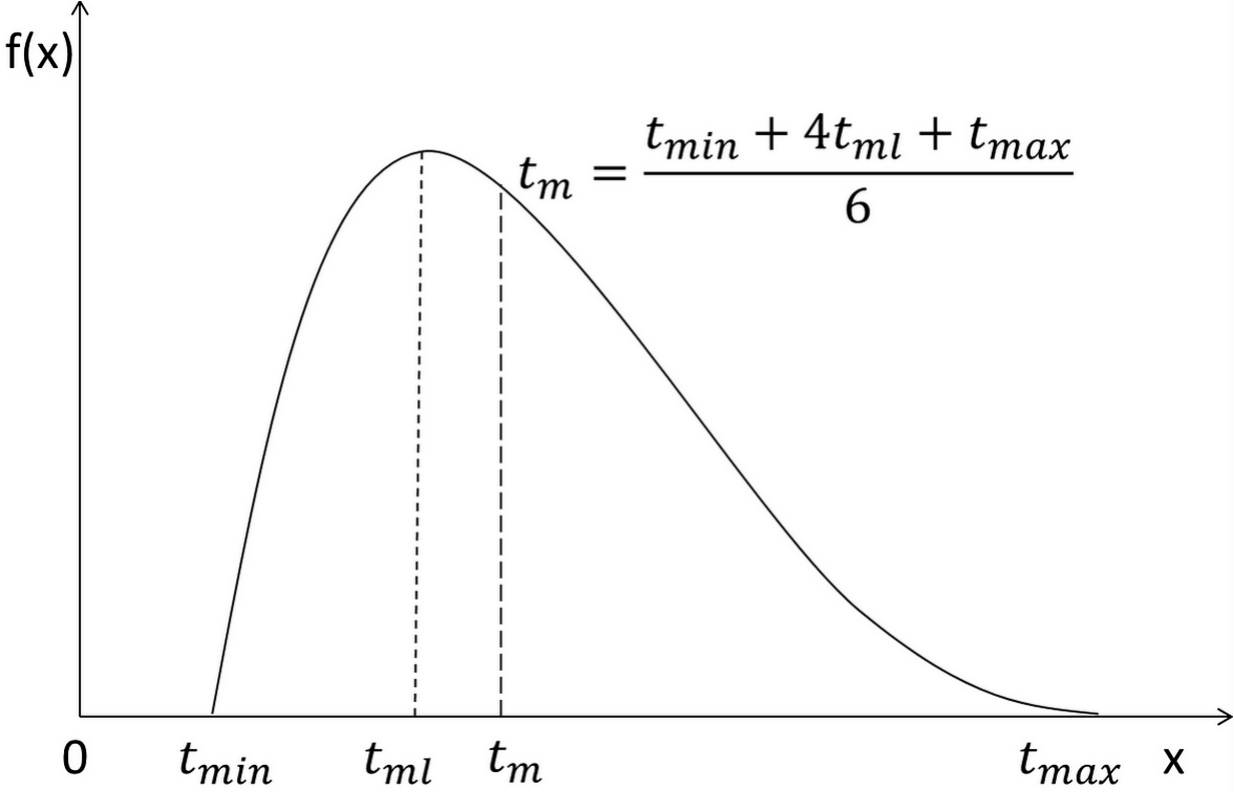
Example of density function of the PERT-beta distribution.

The estimation of the duration of each activity is the key factor in the PERT/CPM. In Fig 2, the durations are deterministic. It is rare to have such certainty in practice. The PERT/CPM would not have any practical value if it could not give a convincing method to estimate the durations of every activity. The PERT defines stochastic variables on activity durations which are assumed as independent to each other. Eqs 1 and 2 give the probabilistic duration and variance of each activity respectively. The distribution of the activity durations is assumed to follow a PERT-beta distribution (Fig 4), which is derived from the Beta distribution (normally given by the probabilistic density function Eq 3).

$$t_{m}=\frac{t_{min}+4t_{ml}+t_{max}}{6}$$ (1)

$$v^{2}={\left(\frac{t_{max}-t_{min}}{6}\right)}^{2}$$ (2)

Where:

t_m_−expected time (mean)

v–variance of duration

t_min_−optimistic time (the possible shortest time)

t_ml_−most likely time estimate (the most frequently occurred time)

t_max_−pessimistic time estimate (the possible longest time)

$$f\left(x|a,b\right)=\frac{\Gamma \left(\alpha +\beta \right)}{\Gamma \left(\alpha \right)\Gamma \left(\beta \right)}\frac{{\left(x-a\right)}^{\alpha -1}{\left(b-x\right)}^{\beta -1}}{{\left(b-a\right)}^{\alpha +\beta -1}}\;,\;\alpha ,\beta >0$$ (3)

Where:

a, b–the endpoints of the domain of x (0 and 1 in the standard Beta distribution),

α, β–the shape parameters of the Beta distribution.

The PERT-beta distribution is constructed by identifying $\alpha =1+\lambda \frac{t_{ml}-t_{min}}{t_{max}-t_{min}}$ and $\beta =1+\lambda \frac{t_{max}-t_{ml}}{t_{max}-t_{min}}$. λ is the scale parameter which is default 4 in the PERT-beta distribution [9]. α>1 and β>1 ensures f(x) has one maximum and trends to zero towards the endpoints t_min_ and t_max_. Instead of the domain (0,1) in the standard Beta distribution, it can be rescaled for variables between a and b by applying x = a + B(α,β) ∙ (b − a). Outside of the domain of x, f(x) = 0.

It has been a common standard to assume that the activity durations follow the PERT-beta distribution for several reasons. First and most important, the PERT-beta distribution is found more realistic than the other types of distributions, after investigating the shapes of all the other distributions. The PERT-beta distribution is continuous, but without the predetermined shape. It enables skewing the shape based on the time estimates given. The duration is not negative, and the PERT-beta distribution can only have positive domain, unlike some other distribution (e.g. normal distribution). The values of the probabilistic density at the endpoints of the domain of the PERT-beta distribution are zero, instead of tending to infinity. Furthermore, the three time estimates (optimistic, most likely, and pessimistic time) are indeed available in practice, and the mean and variance can be approximated by using the three time estimates. Additionally, the easy input requirements relatively boost the prevalence of the PERT-beta distribution, rather than others.

### 2.2 Discussion and Modification of PERT/CPM for using in Underground Mining Operations

#### 2.2.1 Discussion of PERT/CPM for using in Underground Mining Operations

After the debut of CPM and PERT for more than five decades, there have been new techniques developed based on PERT/CPM, which are however more sophisticated for mining operators and require complex user input. The PERT/CPM are particularly appropriate tools to be used in the scheduling for discrete events, e.g. serial operations in underground mining (including scaling operation, shotcreting operation, bolting operation, drilling operation, explosive charging operation). Nonetheless, some drawbacks in both techniques have restricted their applications in underground mining operations. First, the PERT/CPM do not give a method to find the predefined sequence. It is normally complicated to determine the sequence of many mining operations for many working faces. There has been a study for solving this problem [10]. Next, the time estimates are subjective, based on the personal knowledge and abilities. This cannot guarantee the objectiveness of the input and take advantage of the data recorded in mining management system. Moreover, it is required to estimate the duration for every operation. There can be hundreds of operations to be carried out in large-scale underground mine, and the operations can vary considerably. It is almost impossible and inefficient to estimate all the durations manually. Additionally, the PERT analysis assumes the mean *t*_*m*_ and variance *v* are normally distributed, because it is rather difficult to calculate the cumulative probability function of the PERT-beta distribution. The probability of the completion time of the entire mining process can be obtained by summing the means and variances on the critical path, and then the probability of the completion time can be obtained according to the standard normal distribution table [11]. This assumption relies on the central limit theorem, which requires sufficient amount of operations. In underground mining operations, it is quite doubtful whether there are sufficient operations on the critical path. Therefore it cannot warrantee the assumed normal distribution, and the probability of the complete time is questionable. Furthermore, the CPM neglects the existence of different probabilities of the mining operations’ completion time. For example, it calculates the critical path simply using the deterministic values of the completion time of different activities, though there are different probabilities behind these deterministic values. This can lead to a wrong critical path. In view of the above disadvantages, a new method should be customized for using in underground mining, based on the PERT/CPM and combining other theories.

#### 2.2.2 Modification based on PERT/CPM for using in Underground Mining Operations

The new approach should overcome the above drawbacks existing in the PERT and CPM, in order to be used for underground mining operations. Unlike the conventional time-estimating method in the PERT, this new method utilizes the probabilistic density function of underground mining machines’ working rates, estimates the durations and calculates the probability of durations accordingly. Compared with the other probabilistic distributions, the PERT-beta distribution is also suitable for the probabilistic distribution of the working rates. The main reason is that the shape of the PERT-beta distribution is more realistic for working rate, compared to other probabilistic distributions. The shape of the PERT-beta distribution is convex, and can be asymmetric by being skewed right or left. The PERT-beta distribution is bounded within a positive and finite domain while some other distributions have either infinite or negative domains. Based on the variety of the types of rock, mining machines normally have two boundaries of their working rates. The lower boundary represents the minimum working rate, while the higher boundary represents the maximum working rate. Additionally, the PERT-beta distribution can be approximated by using Triangular distribution which is normally given by the probabilistic density function in Eq 4 and the cumulative distribution function in Eq 5. This makes it possible to calculate the probability of activity duration by integration instead of being estimated by Monte-Carlo simulation. It has been concluded that the usage of the Triangular distribution instead of the PERT-beta distribution does not result in significant differences from a practical point of view [12]. In fact, the precision of the three point estimation is much more crucial in determining the distribution.

$$\text{f}\left(\text{x}|\text{a},\text{c},\text{b}\right)=\{\begin{array}{c} \text{f}\left(\text{x}|\text{a},\text{c}\right)=\frac{2\left(\text{x}-\text{a}\right)}{\left(\text{b}-\text{a}\right)\left(\text{c}-\text{a}\right)}\\ \text{f}\left(\text{x}|\text{c},\text{b}\right)=\frac{2\left(\text{b}-\text{x}\right)}{\left(\text{b}-\text{a}\right)\left(\text{b}-\text{c}\right)} \end{array}$$ (4)

$$\text{F}\left(\text{x}|\text{a},\text{c},\text{b}\right)=\{\begin{array}{c} \text{F}\left(\text{x}|\text{a},\text{c}\right)=\frac{{\left(\text{x}-\text{a}\right)}^{2}}{\left(\text{b}-\text{a}\right)\left(\text{c}-\text{a}\right)}\\ \text{F}\left(\text{x}|\text{c},\text{b}\right)=1-\frac{{\left(\text{b}-\text{x}\right)}^{2}}{\left(\text{b}-\text{a}\right)\left(\text{b}-\text{c}\right)} \end{array}$$ (5)

Where:

a, b–the endpoints of the domain of x,

c–the mode, which appears most frequently, i.e. x-ordinate of the peak.

The following gives the probabilistic density function (Eq 9) and cumulative distribution function (Eq 10) of activity duration customized for underground mining operations, where y is the duration, k is the workload, a is the minimum working rate, b is the maximum working rate, and c is the mode of the working rates. Assuming it requires *n* independent serial operations on a workface, x_i_ is the predicted or instant work rate at Face i, y_i_ is the duration of single operation of x_i_, and F(y_i_)is the probability of that duration y_i_, hence, the total duration for working on this workface is $\sum_{1}^{n}y_{i}$, and the probability of that total duration is $\prod_{1}^{n}F(y_{i})$. Similarly, if y_i_ is the duration of an operation in the critical path, then the total duration of the entire process is ∑*y*_*i*_, and the probability of completing the entire process with in that total duration is ∏*F*(*y*_*i*_).

$$\text{Given}\;\text{X}=\left\{\text{x}:{\text{f}}_{\text{X}}\left(\text{x}\right)>0\right\}\;\text{and Y}=\left\{\text{y}:\text{y}=\text{g}\left(\text{x}\right)\;\text{for x}\in \text{X}\right\}$$ (6)

**Theorem** Let X have probabilistic density function f_X_(x) and let Y = g(X), where g is a monotone function. Let X and Y be defined by (6). Suppose that f_X_(x) is continuous on X and that g^-1^(y) has a continuous derivation on Y. Then the pdf of Y is given by:

$${\text{f}}_{\text{Y}}\left(\text{y}\right)={\text{f}}_{\text{X}}\left({\text{g}}^{-1}\left(\text{y}\right)\right)\left|\frac{{\text{dg}}^{-1}\left(\text{y}\right)}{\text{dy}}\right|\;\text{y}\in \text{Y}$$ (7)

**Theorem** Let X have cumulative distribution function F_X_(x), let Y = g(X), and let X and Y be defined in (6). If g is a decreasing function on X and X is a continuous random variable, then

$${\text{F}}_{\text{Y}}\left(\text{y}\right)={\text{1-F}}_{\text{X}}\left({\text{g}}^{\text{-}1}\left(\text{y}\right)\right)\;\text{y}\in \text{Y}$$ (8)

Hence, given *y = k/x* (x, y, k>0) as stated in (6), the probabilistic density function and cumulative distribution function of activity duration are obtained as below:

$$\text{f}\left(\text{y}|\frac{\text{k}}{\text{b}},\frac{\text{k}}{\text{c}},\frac{\text{k}}{\text{a}}\right)=\{\begin{array}{c} \text{f}\left(\text{y}|\frac{\text{k}}{\text{b}},\frac{\text{k}}{\text{c}}\right)=\frac{2\left(\text{b}\frac{\text{k}}{{\text{y}}^{2}}-\frac{{\text{k}}^{2}}{{\text{y}}^{3}}\right)}{\left(\text{b}-\text{a}\right)\left(\text{b}-\text{c}\right)}\\ \text{f}\left(\text{y}|\frac{\text{k}}{\text{c}},\frac{\text{k}}{\text{a}}\right)=\frac{2\left(\frac{{\text{k}}^{2}}{{\text{y}}^{3}}-\text{a}\frac{\text{k}}{{\text{y}}^{2}}\right)}{\left(\text{b}-\text{a}\right)\left(\text{c}-\text{a}\right)} \end{array}$$ (9)

$$\text{F}\left(\text{y}|\frac{\text{k}}{\text{b}},\frac{\text{k}}{\text{c}},\frac{\text{k}}{\text{a}}\right)=\{\begin{array}{c} \text{F}\left(\text{y}|\frac{\text{k}}{\text{b}},\frac{\text{k}}{\text{c}}\right)=\frac{{\left(\text{b}-\frac{\text{k}}{\text{y}}\right)}^{2}}{\left(\text{b}-\text{a}\right)\left(\text{b}-\text{c}\right)}\\ \text{F}\left(\text{y}|\frac{\text{k}}{\text{c}},\frac{\text{k}}{\text{a}}\right)=1-\frac{{\left(\frac{\text{k}}{\text{y}}-\text{a}\right)}^{2}}{\left(\text{b}-\text{a}\right)\left(\text{c}-\text{a}\right)} \end{array}$$ (10)

**Fig 5:**
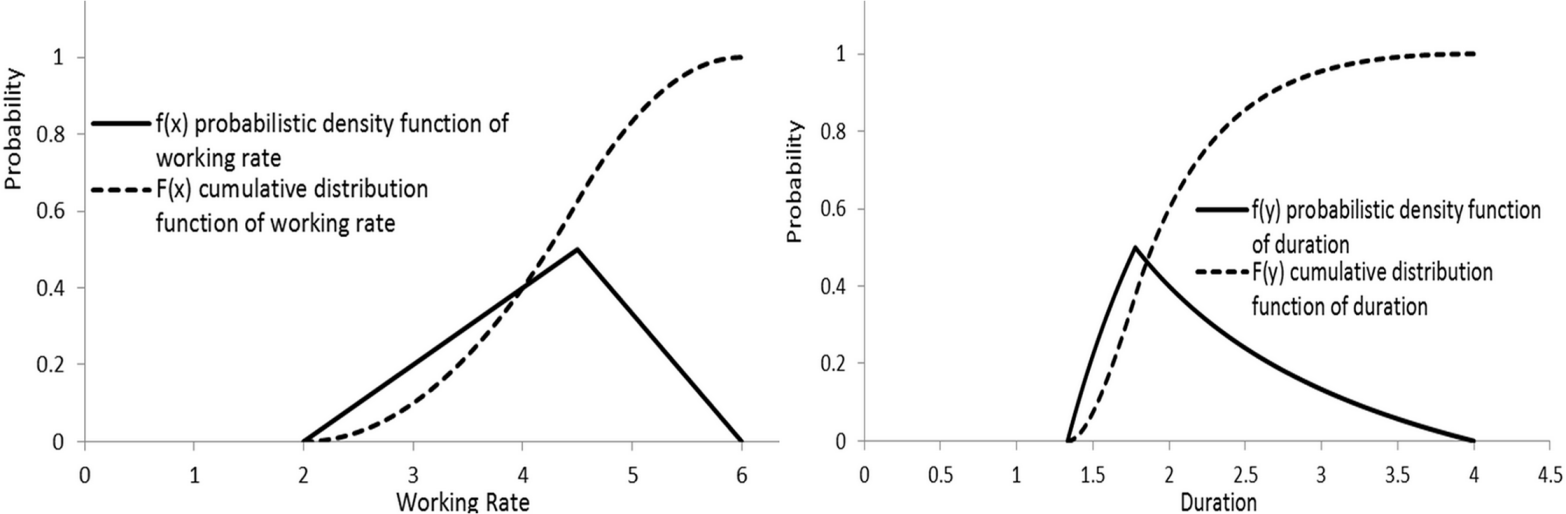
Examples of the probabilistic density and cumulative distribution functions of working rate (*f(x)* and *F(x)*) and duration (*f(y)* and *F(y)*).

This new method can use machines’ data acquired from experiments and/or historic record, which prevents subjective assumption of time estimate. Additionally, it calculates the durations of mining activities efficiently, directly by using the working rates and workloads, instead of manually estimating the durations one by one. Furthermore, it calculates the probability of the duration of each activity and even for the entire process directly from the cumulative distribution function, without relying on the assumption of normally distributed durations and using the Monte-Carlo simulation. Besides, it can calculate the critical path based on the same probability of the duration for each activity, which highly increases the precision of the critical path. Fig 5 shows examples of the probabilistic density function and cumulative distribution function of working rate in triangular distribution, and their corresponding probabilistic density function and cumulative distribution function of duration.

**Fig 6:**
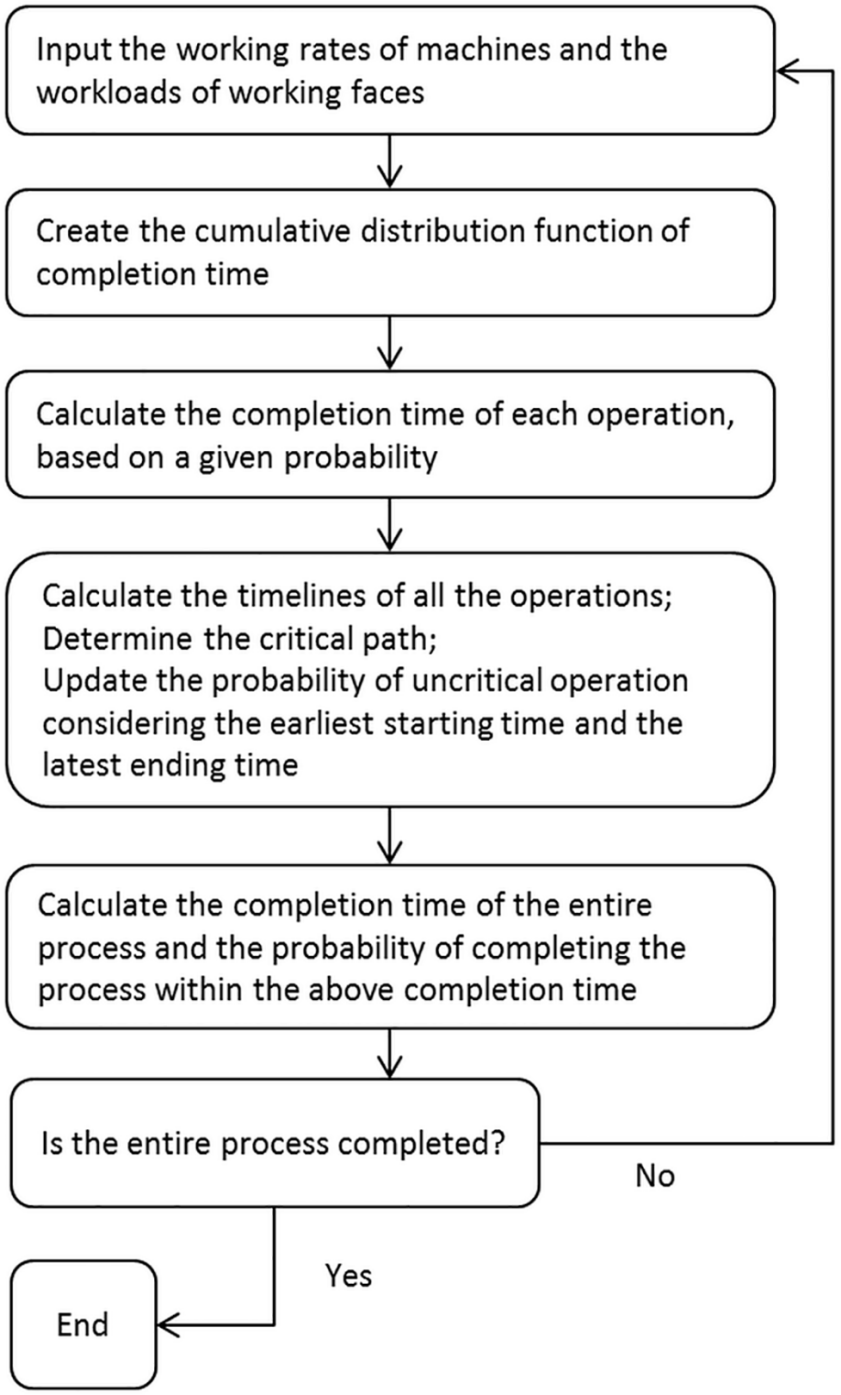
Flow chart of the new algorithm developed based on PERT/CPM.

Fig 6 illustrates the procedure of using the new method. First, it is required to obtain the workload of each operation. The data can be obtained directly from the spreadsheet of a work plan. It is also required to obtain the working rates of each operation, i.e. the minimum working rate, the maximum working rate, and the mode of working rate which is the most frequently appearing working rate. They can be obtained from historic record, machine manufacturer’s manual, experiment, or even skilled operator’s estimation. Next, the probabilistic density function and cumulative distribution function of the completion time of the mining operation can be created. With this cumulative distribution function, the probability of completing that operation within certain duration can be found. On the other side, if the probability of completing an operation within certain duration is given, then the expected duration of an operation can also be calculated. Finally, the total duration of the entire process can be obtained by adding all the durations of the activities in the critical path, and the probability of the entire process can be obtained by multiplying all the probabilities of the activities.

## Algorithm for Mucking Operation of Underground Mining

**Fig 7:**
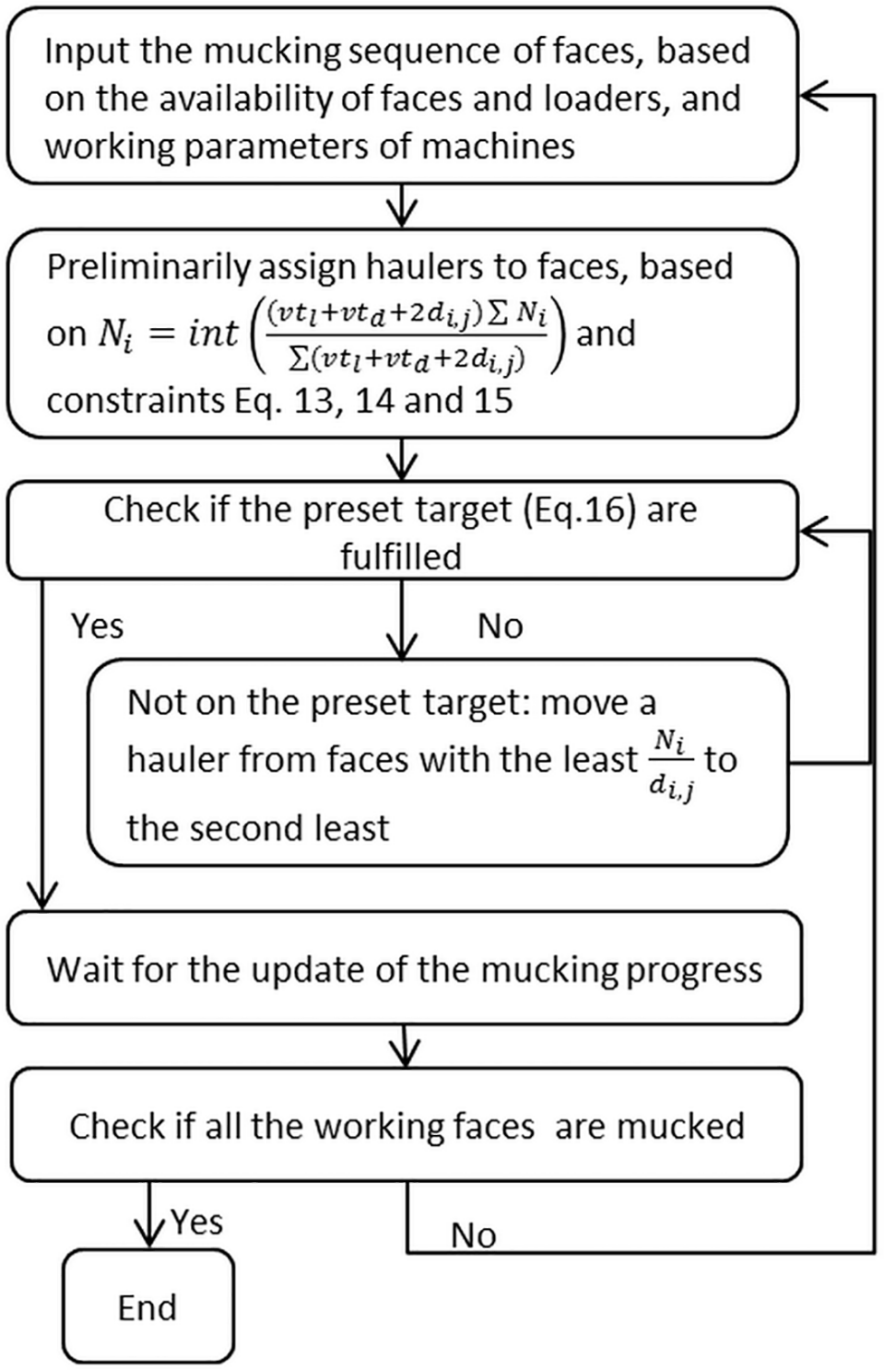
Flow chart of the mucking algorithm.

Mucking operation is a cyclic operation consisting of more than one machine, unlike the serial operation with one single machine. For example one face can only allow one driller, one charger to work at it, but allow one or more mucking machines (i.e. trucks and loaders) in the mucking operation. Mucking operation is used to load, haul and dump the ore from production and excavated waste rock from mine development. The operating time of the mucking operation at a face is varied, and depends mainly on the amount of mucking machines assigned at that face. The previous studies of mucking operation for underground mining focus on equipment selection, fleet management, real-time dispatching, route planning, and self-navigating vehicles. In a long term, the amount/type of loader/truck should be optimized to achieve production targets. The vehicle utilization can be improved, and the queue time can be reduced, using different real-time dispatching policies. However, there is a shortfall between equipment selection and dispatching, i.e. how to assign loader/truck at different working faces. A short-term scheduling model was developed by Nehring et al. [13], which links long-term planning with real-time dispatching. It is specifically designed for sublevel stoping in a conceptual mine. Its objective function is to minimize deviation of production targets for each shift across an entire scheduling period. The solution was obtained using mixed integer-linear programming in approx. 1 min in a common desktop. However, the size of the model is not big enough for real mining practice: it has 5 Load-Haul-Dump loaders (LHD) and 3 trucks being scheduled on 16 stopes for 2 months (120 shifts). Therefore, the rapidness of this scheduling model still needs to be further validated. Additionally, the objective function can lead to relatively low utilization of equipment, because the production is required to reconcile with the pre-set target. Once the production reaches its target, it cannot increase further, even if there are more machines available. Moreover, the model is quite complicated and needs to be coded in CPLEX10.3 which is an IBM-developed commercial software application for solving mathematical models but is not commonly used in mines. Therefore, the application and implementation of the model is relatively challenging for mine operators. It is demanded to propose a more practical and agile method. The mucking algorithm (Fig 7) proposed in this paper aims to minimize the deviation of mucking rate (Eq 12) at working faces, and control the mucking rate above the pre-set target (Eq 15). In order to minimize the deviation of the mucking rates at different working faces R_i,j_, every N_i_/(vt_l_+vt_d_+2d_i,j_) of R_i,j_ should be approximate to each other. In this way, it keeps the time interval between haulers as equal as possible, which ensures smooth traffic in underground mining. There are normally two types of mucking operation at working face:

1. Only one Load-Haul-Dump loader works at the working face. It is in charge of loading ore at the working face, hauling between the working face and its dumping site, dumping at the dumping site, and return for the next cycle.
2. One loader works together with one or more trucks. The loader only loads ore to trucks at the working face. The truck hauls between the working face and the dumping site, dumps the material, and returns for the next cycle.

The following assumptions are applied for the mucking operation:

1. Loaders and trucks are respectively identical (which is actually in favor of fleet management and maintenance);
2. One face has one loader to work;
3. Trucks only dump to the nearest dump (which minimizes fuel consumption);
4. Loading time, dumping time, velocity and payload are constant;

There are basic numerical relations in the mucking operation:

The duration of mucking operation at one working face

$${\text{T}}_{\text{i}}=\frac{{\text{M}}_{\text{i}}\left({\text{vt}}_{\text{l}}+{\text{vt}}_{\text{d}}+2{\text{d}}_{\text{i},\text{j}}\right)}{{\text{N}}_{\text{i}}{\text{M}}_{\text{l}}\text{v}}$$ (11)

The mucking rate at that working face

$${\text{R}}_{\text{i},\text{j}}=\frac{{\text{N}}_{\text{i}}{\text{M}}_{\text{l}}\text{v}}{\left({\text{vt}}_{\text{l}}+{\text{vt}}_{\text{d}}+2{\text{d}}_{\text{i},\text{j}}\right)}$$ (12)

There are also constraints in the mucking operation:

The amount of haulers at one working face should not result in queueing

$${\text{N}}_{\text{i}}\leq \text{int}\left(\frac{{\text{vt}}_{\text{l}}+{\text{vt}}_{\text{d}}+2{\text{d}}_{\text{i},\text{j}}}{{\text{vt}}_{\text{l}}}\right)$$ (13)

The amount of haulers at one dumping site should not result in queueing

$${\text{N}}_{\text{j}}\leq \text{int}\left(\frac{{\text{n}}_{\text{j}}{\text{vt}}_{\text{l}}+{\text{n}}_{\text{j}}{\text{vt}}_{\text{d}}+2\sum_{1}^{{\text{n}}_{\text{j}}}{\text{d}}_{\text{i},\text{j}}}{{\text{vt}}_{\text{d}}}\right)$$ (14)

Dumping rate at a dumping site should not exceed its receptive capacity

$$\sum {\text{R}}_{\text{i},\text{j}}=\sum \frac{{\text{N}}_{\text{i}}{\text{M}}_{\text{l}}\text{v}}{\left({\text{vt}}_{\text{l}}+{\text{vt}}_{\text{d}}+2{\text{d}}_{\text{i},\text{j}}\right)}\leq {\text{D}}_{\text{j}}$$ (15)

The total mucking rate should not be less than the pre-set minimum target

$${\mathrm{\Sigma D}}_{\text{i,j}}\geq {\text{P}}_{\text{target}}$$ (16)

Where:

R_i,j_−mucking rate at Working Face I assigned for Dumping Site j

P_min_−pre-set minimum mucking rate

M_l_−payload of truck or LHD loader

v–velocity of truck or LHD loader

N_i_−the amount of haulers at Working Face i, integer

N_j_−the amount of haulers at Dumping Site j, integer

n_j_−the amount of working faces assigned for Dumping Site j, integer

d_i,j_−the distance between Face i and its Dump j

t_l_−loading time

t_d_−dumping time

T_i_−mucking time at Face i

M_i_−ore mass at Face i

D_j_−the receptive capacity at Dump j

This algorithm can be coded in a stand-alone software application which can facilitate its implementation for the mucking operation in underground mining practice. It has an agile structure and simple constraints which can ensure the rapidness of obtaining the solution. Furthermore, this algorithm minimizes the deviation of mucking rates at different working faces under the constraints (Eqs. 13 14 and 15), and controls the mucking rate. The minimum deviation of mucking rates implies that N_i_/(vt_l_+vt_d_+2d_i,j_) of each face is approximated with the others. This can averagely distribute haulers on route to avoid traffic conflict, and theoretically ensure the distances between trucks are longest. Additionally, since the mucking rate at each working face is similar, the risk of lack of ore supply can be reduced, in case that some faces are unexpectedly inaccessible for short period. Unlike the algorithm developed by Nehring, the objective function does not reduce the utilization of machines, because this algorithm allows the actual productivity to be greater than the pre-set target (Eq 16).

## Application Based on the Data from Kittilä Mine

Kittilä mine is an underground gold mine located in Lapland, Finland. This mine is owned by Agnico Eagle Mine Limited. Its production started in 1 May 2009. The mine produces around 3,000 tonnes of ore per day and it is targeting to produce 4,300 kilograms of gold in 2014. The average production is planned to be roughly 4,700 kilograms of gold per year in 2015 to 2016. Kittilä mine started as two open pits which are called Suuri and Roura. The underground mine was developed and started operating in October 2010. Both open pits had already been mined out in November 2012. Now only the underground mining exists. The excavated ore is hauled by truck to the surface and dumped at ore stockpile. The stockpile will feed the crusher which is next to the processing plant. In Kittilä mine, two types of equipment fleet are used, i.e. the development (excavation) fleet and the production fleet. The development fleet is used for constructing tunnels and openings in the underground, like access ramp and drifts. The production fleet is used to mine out ore from the ore body.

The underground mining operations run according to the weekly plan. There are weekly plans for mine development and production respectively. The preparation of the weekly plan starts from the longest horizon of the mine plan, which is the Life-Of-Mine (LOM) plan that can vary from 10 to 50 years. From the LOM plan, 18-month plan is derived. After that, monthly plan is created according to the 18 months plan, and finally the weekly plan is created based on the monthly plan. The weekly plan is implemented in the mine by communicating it between foreman and his crew. These foremen usually use their own experience and personal judgement to implement the plan and to handle unexpected events during the implementation. There has been a schedule optimizer of underground mobile mining equipment described and developed by Song et al (2014). It can assist the foreman to make an optimized schedule of mining equipment and reschedule quickly against unexpected events. However, the foreman still needs the information of the work in progress, e.g. the critical operations, the probabilities of completion time. With this knowledge, the foreman can be more confident during the shift, obtain more accurate expectation to handle the crew and the operations, and have a better overview of the shift. Additionally, the foreman needs to optimally assign mucking tasks to the mucking machines, in order to achieve the pre-set development or preproduction target. This will also let the foreman take better advantage of the underground positioning and dispatching systems, if there are any.

The mine development is normally more complicated than mine production, and comprise of more operations, therefore the mine development was chosen as the example in the case study. The data of several shifts of a weekly plan of mine development were used, and the machines’ data were obtained from manufacturers’ manuals or estimated by skilled operators. The weekly plan data mainly showed the headings which should be excavated during the week, and the machines’ data are mainly the working parameters of the machines.

The shifts (2 days, 48 hours) were in the weekly plan during the period of 4–10 September 2013. In these shifts, three working faces, Working Face 4 (WF4), Working Face 3 (WF3), and Working face 5 (WF5) were excavated in sequence, by one set of mining equipment. The excavation at each working face included the serial operations of scaling, cleaning, shotcreting, bolting, drilling, charging and dust suppression. Table 1 shows the operation information using the conventional method, including the working time of each mining machine at every working face, the time difference between the Earliest Starting Time (EST) and Latest Ending Time (LET), and the probability of completing within the time difference. The working time was derived from the workload for each machine at each working face divided by the most frequent working rate. EST and LET were obtained according to the Critical Path Method. The probability of completing between EST and LET was obtained according to Eq 10 The critical path is also indicated in Table 1. It is noted that the probability of the completion time is varied in every operation, and the entire process would be completed in 30.5 hours with a confidence of only 5.5%. Table 2 shows the same operation information as in Table 1. Unlike the procedure used in Table 1, the suggested method first sets a desired probability to 90%. Then, it calculates the related working time. All the critical operation has a confidence of 90%, and the uncritical operation has a confidence above 90%. It is noted that the critical path is changed in Table 2, and the entire process can be completed in 47.5 hours, which is close to the actual time of 48 hours, with a confidence of 49%. If the confidence is set at 99% for each operation, it can be found that the entire process can be completed in 78 hours with a confidence of 93%.

**Table 1:** Probability and critical path using the conventional method.

	Scaler	Cleaner	Shotcreter	Bolter	Driller	Charger	Dustsprayer
WF4	In critical path	In critical path	In critical path	In critical path	In critical path		
Working time (hour)	3.83	2	1.91	5.63	6	2.90	1.40
Time difference (hour) between EST and LET	3.83	2	1.91	5.63	6	6.10	6.14
Probability of completing between EST and LET	15%	50%	30%	50%	50%	100%	100%
WF3					In critical path		
Working time (hour)	3.83	2	1.91	5.63	6	2.90	1.40
Time difference (hour) between EST and LET	6	4.17	4.09	6	6	3	1.55
Probability of completing between EST and LET	100%	100%	100%	78%	50%	88%	97%
WF5					In critical path	In critical path	In critical path
Working time (hour)	1.91	1	0.96	2.81	3	1.45	0.70
Time difference (hour) between EST and LET	12.94	11.94	10.98	6.38	3	1.45	0.70
Probability of completing between EST and LET	100%	100%	100%	100%	50%	78%	50%

**Table 2:** Probability and critical path using the new method.

	Scaler	Cleaner	Shotcreter	Bolter	Driller	Charger	Dustsprayer
WF4	In critical path	In critical path	In critical path	In critical path			
Working time (hour)	5.02	2.13	2.11	13.08	6.48	3.03	1.51
Time difference (hour) between EST and LET	5.02	2.13	2.11	13.08	13.15	13.36	13.37
Probability of completing between EST and LET	90%	90%	90%	90%	100%	100%	100%
WF3				In critical path			
Working time (hour)	5.02	2.13	2.11	13.08	6.48	3.03	1.51
Time difference (hour) between EST and LET	13.08	10.19	10.17	13.08	6.54	3.30	1.79
Probability of completing between EST and LET	100%	100%	100%	90%	93%	99%	100%
WF5				In critical path	In critical path	In critical path	In critical path
Working time (hour)	2.51	1.07	1.06	6.54	3.24	1.52	0.75
Time difference (hour) between EST and LET	23.27	21.82	21.14	6.54	3.24	1.52	0.75
Probability of completing between EST and LET	100%	100%	100%	90%	90%	90%	90%

By using this method the drawbacks of the PERT are eliminated and the probability of each activity is obtained more efficiently in an objective manner. This method also corrects the procedure of determining the critical path which the CPM uses. The results, i.e. the working time and the probability, obtained from the new method are more accurate and more practical than the conventional PERT/CPM.

After the above mentioned operations at every working face, the excavated material should be transported to dumping sites. The algorithm of mucking operation is used to first assign the mucking machines (loaders and trucks) to every working faces, and re-assign the machines upon unexpected events. Based on the data of working faces and mucking machines (incl. 3 loaders and 7 trucks), the initial assignment of mucking machines are demonstrated in Table 3. Working Faces 4, 3 and 5 were available at 23:57, 5:40 and 6:37 respectively. The mucking rate was around 140t/h. It is noted that when there is another working face available or completed, the algorithm will re-assign the trucks among the available working faces.

**Table 3:** Assignment of mucking machines at working faces.

Working Face ID	Starting Time	Ending Time	Truck Amount	Workload (ton) Done	Workload (ton) Left
4	23:57	5:40	7	756	144
4	5:40	6:37	3	81	63
3	5:40	6:37	4	108	792
4	6:37	8:05	2	63	0
3	6:37	8:05	3	96.08	695.93
5	6:37	8:05	2	65.1	384.9
3	8:05	12:21	4	510	185.93
5	8:05	12:21	3	384.9	0
3	12:21	13:14	7	185.93	0

**Fig 8:**
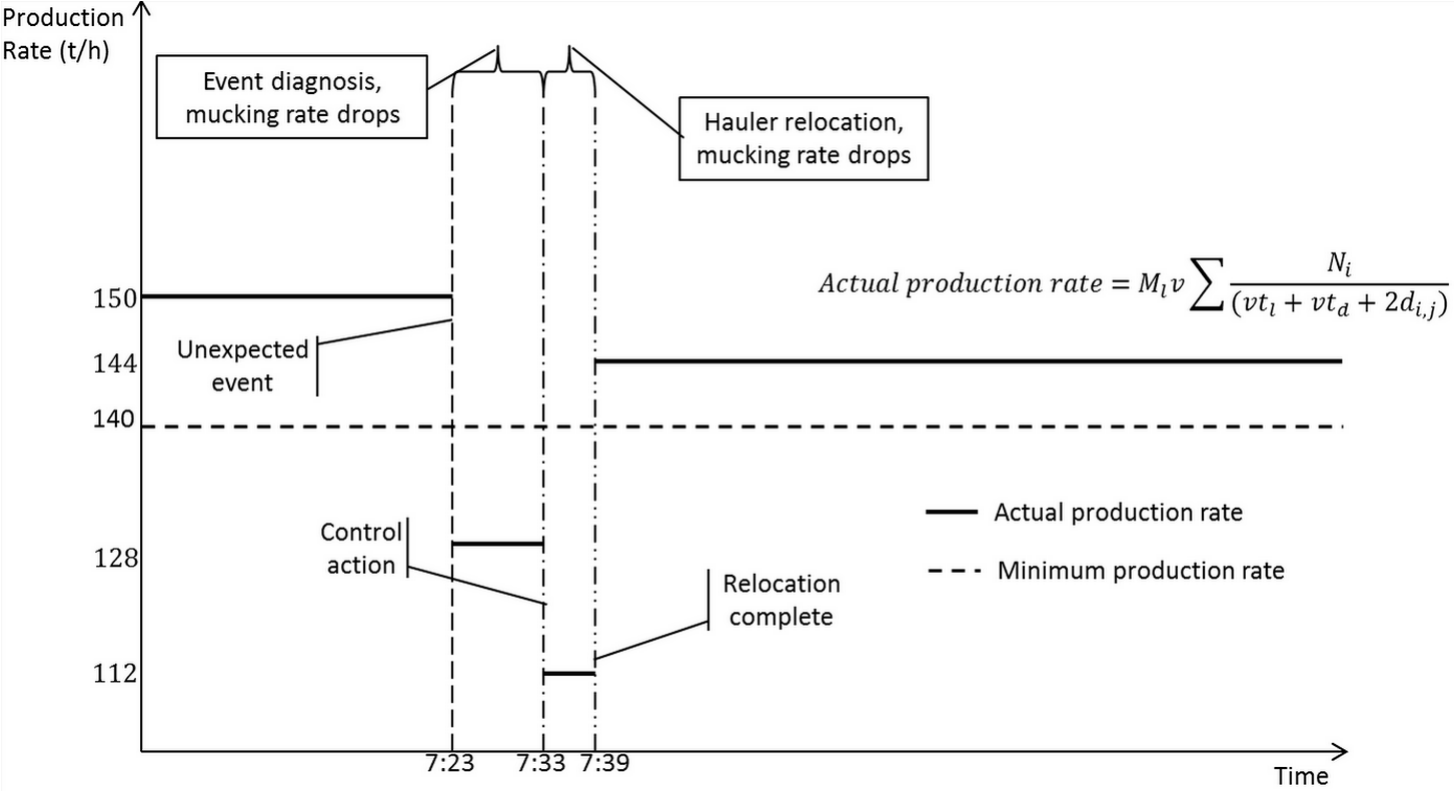
Re-assign the trucks upon an unexpected event.

Fig 8 demonstrates another application of the algorithm of mucking operation. It is a simulated result of re-assigning the mucking machines when encountering an unexpected event. A scenario of an unexpected event was created: a truck of Working Face 4 was broken down at 7:23. The target of mucking rate was set to 140t/h, and the mucking rate was around 150t/h before time 7:23. At time 7:23, a truck of Working Face 4 was broken down, which caused a drop of mucking rate. From 7:23 to 7:33, the event was investigated. At time 7:33, a control action was decided to be implemented. By using the algorithm of mucking operation, it gives the proposed solution: a truck from Working Face 5 was transferred to Working Face 4 to compensate the loss of mucking rate. From 7:33 to 7:39, because of the relocation of the truck, the mucking rate dropped further. Furthermore, after the truck was relocated, the mucking rate was resumed to 142t/h above the target.

## Conclusions and Recommendations for Future Work

This paper presents an approach to process control for underground mining operations. It analyzes the existing PERT/CPM and modifies the methods according to the practice of underground mining. The new algorithm modified from the PERT/CPM can efficiently and accurately estimate the probability of activity and the critical path. This paper also refers to the existing algorithms studied for the mucking operation of underground mining. In addition, it suggests a new algorithm to first assign mucking machines and to rapidly adjust the mucking operation for a specific target under external constraints.

This approach can assist mine managers to achieve the production target and reconcile the actual production, with an innovative and integrated process control method covering the entire operations of development and production. The steps are as follows:

1. Schedule underground mining operations (Song et al, 2014).
2. Determine the critical path of the mining operations and their probabilities of completion time.
3. If an operation is supposed to be delayed, increase the working rate of the related machine. If it is delayed, re-schedule the mining operations to obtain an updated schedule as in Step 1), and determine the critical path as in Step 2).
4. Assign the mucking machines to transport the excavated/mined materials.
5. If there is an unexpected event in the mucking operation, re-assign the mucking machines as in Step 4).

This approach has been coded as a stand-alone software application. In the near future, this approach will be tested and demonstrated in a more complicated case with a longer period and bigger scale of mine production.
